# Catatonia: Closer Than We Think

**DOI:** 10.1192/bjo.2026.11899

**Published:** 2026-06-30

**Authors:** Mohammad Saif Uddin, Prafulla Chandravadiya

**Affiliations:** Devon Partnership NHS Trust, Exeter, United Kingdom

## Abstract

**Aims:**

This is a comparative study between two unusual and distinct cases of catatonia, managed by the Liaison psychiatry team that helped us to broaden our thinking and explore variable differential diagnoses for patients’ management.

**Methods:**

Case1: Mr.P is a 74-year-old male who was admitted following unusual behaviour, including shouting with slurred speech outside his house. During his admission, he was non-verbal, non-compliant with oral intake including medication and demonstrated bradykinesia and stiff joints. His initial investigations were unremarkable.

Mr. P has a background history of Paranoid Schizophrenia and been treated with Aripiprazole 30mg. Four months prior, he had a lengthy hospital admission with a similar presentation, and he was conservatively managed for hypoactive delirium.

Our differential diagnoses included hypoactive delirium, catatonia, CNS infection and NMS. When the physical health issues were ruled out, a Lorazepam challenge test was initiated; this was effective and made a significant change in his presentation. Thus, we diagnosed him with Catatonia and treated him with high dose of Lorazepam according to the policy. At discharge he had returned to his baseline.

**Results:**

Case2: Mrs.Q is a 78-year-old female who was admitted to hospital after concerns of self-neglect and confusion. Her initial investigations showed an AKI and raised inflammatory markers. Thus, she was treated with IV fluid and antibiotics. Collateral from daughter suggested that Mrs. Q had a rapid decline in her presentation prior to the hospital admission.

Mrs.Q has a background history of depression and been treated with Mirtazapine 15mg.

Despite optimisation in her physical health, Mrs. Q did not show significant improvements. She had minimal engagement, minimal oral intake, very slow movements, and significant thought block. Initially, it was presumed that Mrs.Q had a slowly resolving delirium, however as she was not improving as expected so a Lorazepam challenge test was trialled. Mrs Q responded well to the lorazepam challenge, thus, she was diagnosed with catatonia and treated accordingly.

**Conclusion:**

Catatonia is a neuro-psychiatric condition characterised by marked changes in muscle tone or activity that may alternate between extremes of movement deficits (stupor) and excessive movement (excitement). According to DSM-5 and ICD-11, a diagnosis requires the presence of three or more of the 12 clinical features.It can be validated through BFCRS or KCRS. Catatonia is not a disease, but a syndrome associated with various conditions.Its management includes Benzodiazepams, and/or ECT and supportive care.

